# Adverse radiation effect versus tumor progression following stereotactic radiosurgery for brain metastases: Implications of radiologic uncertainty

**DOI:** 10.1007/s11060-024-04578-6

**Published:** 2024-02-05

**Authors:** Mia Salans, Lisa Ni, Olivier Morin, Benjamin Ziemer, Dante P. I. Capaldi, David R. Raleigh, Harish N. Vasudevan, Jessica Chew, Jean Nakamura, Penny K. Sneed, Lauren Boreta, Javier E. Villanueva-Meyer, Philip Theodosopoulos, Steve Braunstein

**Affiliations:** 1 Department of Radiation Oncology, University of California San Francisco (MS, LN, OM, BZ, DPIC, DRR, HNV, JC, JN, PKS, LB, SB), 505 Parnassus Ave, L75, San Francisco, CA 94158 USA; 2 Department of Neurosurgery, University of California San Francisco (DRR, JEVM, PT), San Francisco, USA; 3 Department of Pathology, University of California San Francisco (DRR), San Francisco, USA; 4 Department of Radiology and Biomedical Imaging, University of California San Francisco (JEVM), San Francisco, USA

**Keywords:** Stereotactic radiosurgery, Adverse radiation effect, Brain metastases

## Abstract

**Background:**

Adverse radiation effect (ARE) following stereotactic radiosurgery (SRS) for brain metastases is challenging to distinguish from tumor progression. This study characterizes the clinical implications of radiologic uncertainty (RU).

**Methods:**

Cases reviewed retrospectively at a single-institutional, multi-disciplinary SRS Tumor Board between 2015–2022 for RU following SRS were identified. Treatment history, diagnostic or therapeutic interventions performed upon RU resolution, and development of neurologic deficits surrounding intervention were obtained from the medical record. Differences in lesion volume and maximum diameter at RU onset versus resolution were compared with paired t-tests. Median time from RU onset to resolution was estimated using the Kaplan–Meier method. Univariate and multivariate associations between clinical characteristics and time to RU resolution were assessed with Cox proportional-hazards regression.

**Results:**

Among 128 lesions with RU, 23.5% had undergone ≥ 2 courses of radiation. Median maximum diameter (20 vs. 16 mm, *p* < 0.001) and volume (2.7 vs. 1.5 cc, *p* < 0.001) were larger upon RU resolution versus onset. RU resolution took > 6 and > 12 months in 25% and 7% of cases, respectively. Higher total EQD2 prior to RU onset (HR = 0.45, *p* = 0.03) and use of MR perfusion (HR = 0.56, *p* = 0.001) correlated with shorter time to resolution; larger volume (HR = 1.05, *p* = 0.006) portended longer time to resolution. Most lesions (57%) were diagnosed as ARE. Most patients (58%) underwent an intervention upon RU resolution; of these, 38% developed a neurologic deficit surrounding intervention.

**Conclusions:**

RU resolution took > 6 months in > 25% of cases. RU may lead to suboptimal outcomes and symptom burden. Improved characterization of post-SRS RU is needed.

## Introduction

Brain metastases are common, occurring in up to 30 to 40% of patients with cancer [1]. Their incidence is expected to rise as more effective systemic therapies and high-quality imaging become increasingly available [2]. In recent years, stereotactic radiosurgery (SRS) has supplanted whole-brain radiation as a preferred treatment modality for patients with limited brain metastases due to its ability to spare neurocognitive function without compromising local control or overall survival [3–5]. Indications for SRS continue to expand, with observational data suggesting that SRS may be feasible in patients with up to 10 brain metastases [6]. Multiple phase III clinical trials exploring quality of life and survival outcomes further are currently underway (NCT04804644, NCT03075072).

Adverse radiation effect (ARE), which may entail necrosis, is a late complication of intracranial radiotherapy that typically occurs within 6 to 18 months after treatment [7]. Estimates of its incidence vary from 0% up to 30% [8] due to variations in its definition and challenges associated with pathologic confirmation. Radiologically, ARE typically appears as a ring-enhancing lesion with surrounding T2/FLAIR hyperintensity on magnetic resonance imaging (MRI) and can manifest clinically as headache, nausea, somatosensory deficits, or vision changes, among other symptoms [9]. These features make it difficult to distinguish from tumor progression, which often exhibits similar radiologic and clinical characteristics. Moreover, contrast-enhancing MRI lesions can represent a mixture of both ARE and recurrent tumor, further complicating their differentiation. Apart from surgical resection and laser interstitial thermal therapy (LITT), management of these entities can vary significantly, ranging from corticosteroids or bevacizumab for ARE to systemic therapy or reirradiation for recurrent tumor. ARE also resolves spontaneously without intervention in up to 50% of cases [10] and is therefore often managed with observation alone when asymptomatic. Accurate diagnosis of ARE versus tumor progression is therefore of critical importance.

Pathologic assessment is the gold standard in diagnosing ARE versus tumor progression; however, this is rarely performed in practice to avoid operative morbidity. Optimal techniques to distinguish ARE from tumor progression in a non-invasive manner have not yet been established. Studies have explored morphologic MRI [11], MR perfusion [12], MR spectroscopy [13], and positron emission tomography (PET) [14] as potential diagnostic tools, yet many of these techniques are currently limited by poor performance or infeasibility in the routine clinical setting. Instead, radiologic uncertainty (RU) between ARE and progressive tumor is typically monitored for change over time with serial MRIs before a presumed diagnosis is declared and a definitive intervention is recommended. This process can take months, during which time patients may experience lesion growth with edema and mass effect resulting in progressive symptoms and ultimately requiring more intensive therapy. While generally acknowledged, no studies to date have described the ramifications of this diagnostic delay after RU. We sought to characterize the management and clinical implications of RU after SRS for brain metastases among patients presented at a single center, high-volume, multidisciplinary SRS Tumor Board.

## Materials and methods

### Study design and participants

This study was approved by our Institutional Review Board. This was a retrospective cohort study of patients reviewed at the University of California San Francisco (UCSF) multi-disciplinary SRS Tumor Board for RU of ARE versus tumor progression at a site of prior SRS between April 2015 and December 2022. All patients were required to have undergone SRS for brain metastases. Exclusion criteria included incomplete medical records after initial RU and/or loss to follow-up before RU resolution.

### Definitions of RU and RU resolution

The date of RU was defined as the date of the MRI on which a lesion indeterminate for tumor progression versus ARE (i.e., RU) was first described. The date of RU resolution was determined based on review of the medical record and defined as the date of surgical resection for diagnostic purposes in the face of uncertainty about ARE versus tumor progression or the date of definitive intervention after a presumed diagnosis of ARE versus tumor progression was reached, including surgical resection, re-irradiation, initiation of/change in systemic therapy, enrollment on hospice or a clinical trial, or initiation of steroids to treat ARE. If no intervention was performed, RU resolution was designated as the date on which the lesion showed improvement or stability in size as described in clinical notes and radiology reports.

### Patient, tumor, and treatment characteristics

Patient, tumor, and treatment characteristics were obtained from the medical record. Specifically, histology of primary disease, history of prior surgical resection at the site of RU, history of prior permanent seed implant brachytherapy at the site of RU, symptoms at the time of RU, use of steroids or bevacizumab at the time of RU, imaging modalities used to resolve RU, final diagnosis of either ARE or tumor progression, interventions performed at the time of RU resolution, and development of neurological deficits surrounding definitive intervention were recorded. Note that a diagnosis could be reached either radiologically or based on pathology; thus, for lesions diagnosed based on imaging, the diagnosis may not have reflected the true lesion etiology. Lesions diagnosed as a mixture of ARE and tumor were classified as tumor progression. Dosimetric data from all prior courses of radiotherapy to sites of RU were obtained by evaluation of UCSF and outside radiation records.

### Assessment of lesions at most recent course of SRS, time of initial RU, and time of RU resolution

For each patient, MRIs from three timepoints were imported from our institutional picture archiving and communication system (PACS) into MIM (version 7.1.4). Timepoints included a) the date of the most recent SRS course (planning SRS MRI), b) the date on which the question of RU was raised, and c) the date of RU resolution or the closest date prior to RU resolution. The contrast-enhancing lesion of interest was manually contoured on the T1 post-contrast MRI sequence at each timepoint for each patient. The maximum diameter and volume of the contrast-enhancing lesion at each timepoint were calculated in MIM. The T2/FLAIR MRI sequence was used to assess for the presence of edema causing mass effect (i.e., compression, distortion, or displacement or brain parenchyma or ventricles or sulcal effacement) on the dates of RU and RU resolution.

### Statistical analysis

Descriptive statistics were used to summarize patient, tumor, and treatment characteristics at baseline. Differences in median lesion volume (cc) and maximum diameter (mm) at the time of RU versus RU resolution were evaluated with paired samples t-tests. The difference in the frequency of edema causing mass effect between RU and RU resolution within the cohort was evaluated with McNemar’s test. The Kaplan–Meier method was used to estimate the median time from completion of the most recent SRS course to the date of RU and the time from RU to the date of RU resolution (months). Associations between clinical characteristics and time to RU resolution were assessed with univariate and multivariate Cox proportional hazards regression. P-values < 0.05 were considered statistically significant. All statistics were performed in R version (version 4.2.2; available at http://www.r-project.org/).

## Results

### Lesion and treatment characteristics

We identified 128 lesions with RU in 123 patients who were presented at the institutional SRS Tumor Board for RU of ARE versus tumor progression at a prior site of SRS for brain metastasis between April 2015 and December 2022. Complete radiation records were available for 125 lesions. Imaging from the most recent course of SRS, initial RU, and RU resolution was available in 126, 128, and 126 cases, respectively. Baseline lesion characteristics are summarized in Table 1. Primary histology was non-small cell lung cancer in 32.8% of lesions and the most common location was in the frontal lobes (29.7%). The median total prescription EQD2 for all radiation courses prior to RU onset was 43.4 Gy. A metastasis had been previously resected at the site of RU in 28.9% of cases and permanent seed implant brachytherapy had been performed at the site of RU in 3.9% of cases. At the time of RU, 66.4% of patients were on systemic therapy; most were on either targeted therapy (39.1%) and/or immunotherapy (21.9%).

**Table 1:** Baseline patient characteristics

Characteristic	Frequency, n (%) (*n* = 128)
Tumor histology	
Non-small cell lung	42 (32.8)
Breast	39 (30.5)
Melanoma	22 (17.2)
Genitourinary^a^	8 (6.3)
Gastrointestinal^b^	7 (5.5)
Gynecologic^c^	3 (2.3)
Other^d^	7 (5.5)
Tumor location	
Frontal	38 (29.7)
Parietal	21 (16.4)
Temporal	18 (14.1)
Occipital	18 (14.1)
Cerebellum	28 (21.9)
Other^e^	5 (3.9)
Tumor laterality	
Left	62 (48.4)
Right	64 (50.0)
Central	2 (1.6)
Median initial target volume, IQR (cc)	2.73 (0.4–7.1)
Median initial target diameter, IQR (mm)	19.0 (9.0–27.5)
Total number of radiation courses to target	
1	98 (76.6)
2	23 (18.0)
3	7 (5.5)
Median total EQD2 (α/β = 10), IQR (Gy)^f^	43.3 (40.0–50.0)
Total EQD2^f^	
0–50 Gy	92 (71.9)
50–100 Gy	27 (21.1)
≥ 100 Gy	9 (7.0)
Median EQD2 at most recent SRS course (α/β = 10), IQR (Gy)	42.0 (40.0–45.9)
Fractionation at most recent SRS course	
1	87 (68.0)
2	1 (0.8)
3	6 (4.7)
4	1 (0.8)
5	33 (25.8)
Median number of fractions at most recent SRS course, IQR	1 (1–5)
Prior resection of lesion	37 (28.9)
Prior seed implant brachytherapy at target site	5 (3.9)
Median time to RU from most recent SRS course, IQR (months)	10.6 (5.2–16.7)

Abbreviations: IQR, interquartile range; EQD2, equivalent dose in 2 Gy fractions; Gy, Gray; SRS, stereotactic radiosurgery

^a^Includes renal cell (*n* = 5), testicular (*n* = 2), prostate (*n* = 1)

^b^Includes colorectal (*n* = 4), gastroesophageal junction adenocarcinoma (*n* = 1), pancreas (*n* = 1), fibrolamellar hepatocellular carcinoma (*n* = 1)

^c^Includes endometrial (*n* = 1), ovarian (*n* = 2)

^d^Includes salivary (*n* = 2), sarcoma (*n* = 1), thymic carcinoma (*n* = 1), thyroid (*n* = 2), unknown (*n* = 1)

^e^Includes pons (*n* = 1), thalamus (*n* = 1), sellar (*n* = 1), corpus callosum (*n* = 1), basal ganglia (= 1)

^f^Does not include brachytherapy doses for 5 patients who received seed implant brachytherapy

### Lesion characteristics at time of initial RU and RU resolution

**Fig. 1:**
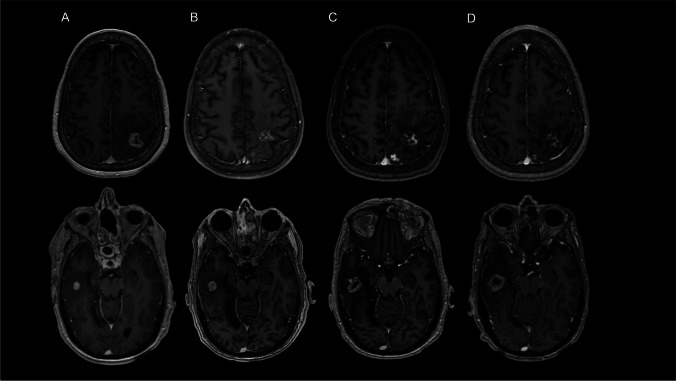
Lesions with RU for representative patients diagnosed with ARE and tumor progression. Lesions with RU on T1 post-gadolinium MRI at **A**) RU onset, **B**) 18 months after RU onset, **C**) 30 months after RU onset, and **D**) RU resolution for representative patients diagnosed with ARE (top panel) and tumor progression (bottom panel)

The median time from the most recent prior course of SRS to RU was 10.6 months. There was no significant difference in the time to RU among lesions ultimately diagnosed as ARE compared with progressive tumor (10 vs. 11 months, *p* = 0.6). Almost half (43.8%) of lesions were associated with symptoms at the time of RU; 41 (32.0%) and 5 (3.9%) required steroids and/or bevacizumab, respectively. The median maximum lesion diameter (20.0 mm vs. 16.0 mm, *p* < 0.001) and median lesion volume (2.7 cc vs. 1.5 cc, *p* < 0.001) were both significantly larger at the time of RU resolution compared with initial RU. A significantly larger proportion of lesions were also noted to result in edema causing mass effect on imaging at RU resolution compared with initial RU (38.3% vs. 21.9%, *p* = 0.001. Lesions with RU on T1 post-contrast MRI at RU onset, 18 and 30 months after RU onset, and RU resolution from two representative patients are demonstrated in Fig. 1.

### Time to RU resolution

**Fig. 2:**
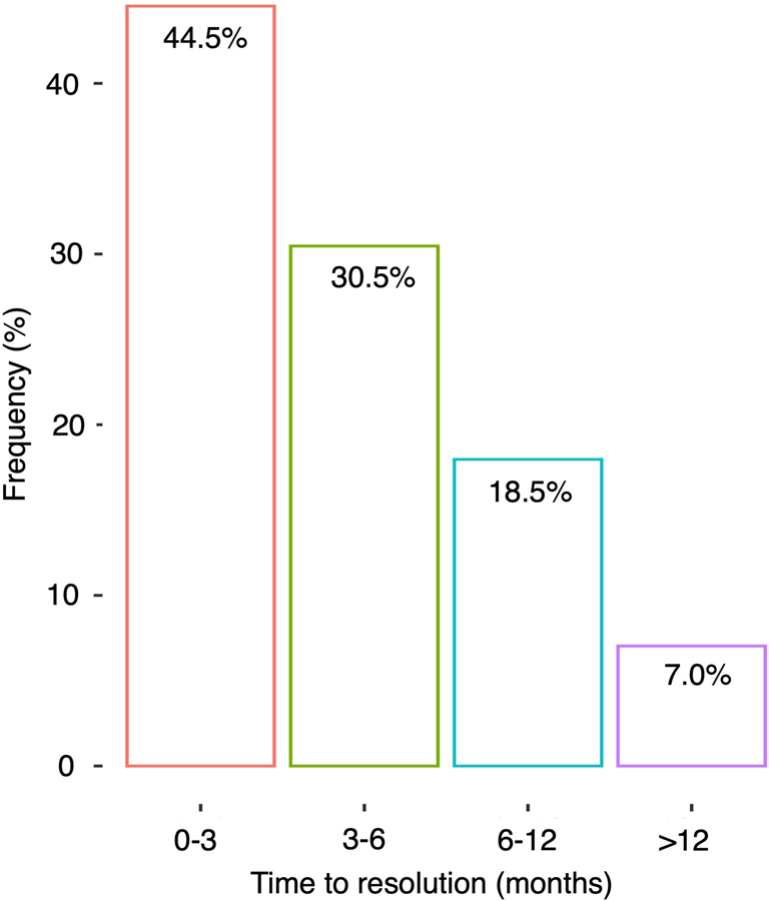
Distribution of times to resolution of radiographic uncertainty

The distribution of times to resolution of RU among all lesions in our cohort is demonstrated in Fig. 2. The median time to RU resolution from the initial date of RU was 3.3 months (IQR: 2.3–6.0). Resolution of RU took between 0 and 3 months in 44.5% of cases; however, it took more than 6 months in 25% of cases and more than 12 months in 7.0% of cases.

Univariate and multivariate associations between patient, treatment, and RU lesion characteristics are shown in Table 2. On univariate analysis, lesions that received a total EQD2 > 100 Gy had significantly shorter time to RU resolution compared to those that received a total EQD2 ≤ 50 Gy (HR = 0.45, *p* = 0.03), but there was no difference in the time to RU resolution between those treated to 50–100 Gy compared to ≤ 50 Gy (HR = 0.96, *p* = 0.86). Larger lesion volume (HR = 1.05, *p* = 0.006) and diameter (HR = 1.02, *p* = 0.01) at the time of RU were associated with longer time to RU resolution. Cases where MR perfusion imaging was used to aid in the resolution of RU had significantly shorter time to RU resolution compared to those that did not (HR = 0.6, *p* = 0.001). Both lesion volume at the time of RU (HR = 1.04, *p* = 0.01) and use of MR perfusion imaging (HR = 0.60, *p* = 0.006) remained significantly associated with time to RU resolution on multivariate analysis. RU lesion diameter was not included in the multivariate analysis due collinearity with lesion volume.

**Table 2:** Univariate and multivariate Cox proportional hazards analyses of time to resolution of radiographic uncertainty

	Univariate			Multivariate		
Predictor	Hazard ratio	95% CI	p-value	Hazard Ratio	95% CI	*p*-value
Total EQD2						
0–50 Gy	Ref	-		Ref	-	-
50–100 Gy	0.96	0.63, 1.48	0.86	0.90	0.58, 1.40	0.64
> 100 Gy	0.45	0.22, 0.91	0.03*	0.54	0.26, 1.11	0.09
Histology at initial treatment						
Other	Ref	-	-			
NSCLC	1.16	0.7, 1.93	0.56			
Breast	0.94	0.57, 1.57	0.82			
Melanoma	1.60	0.89, 2.88	0.12			
Lesion location						
Frontal	Ref	-	-			
Parietal	0.77	0.45, 1.32	0.34			
Temporal	0.72	0.40, 1.28	0.26			
Occipital	1.72	0.97, 3.06	0.06			
Cerebellum	0.63	0.38, 1.02	0.06			
Other	0.46	0.18, 1.17	0.10			
Prior surgery						
No	Ref	-	-			
Yes	0.89	0.76, 1.65	0.56			
RU lesion volume (cc)	1.05	1.02, 1.09	0.006*	1.04	1.01, 1.09	0.01*
RU lesion diameter (mm)	1.02	1.01, 1.04	0.01*			
Systemic therapy at RU						
No	Ref	-	-			
Yes	0.81	0.56, 1.17	0.26			
Targeted and/or immunotherapy at RU						
No	Ref	-	-			
Yes	0.93	0.65, 1.32	0.68			
Mass effect at RU						
No	Ref	-	-			
Yes	0.93	0.61, 1.42	0.75			
Symptomatic at RU						
No	Ref	-	-			
Yes	0.97	0.68, 1.38	0.85			
Bevacizumab at RU						
No	Ref	-	-			
Yes	0.55	0.22, 1.38	0.20			
Steroids at RU						
No	Ref	-	-			
Yes	0.99	0.68, 1.45	0.98			
Diagnosis						
Tumor	Ref	-	-			
ARE	1.21	0.85, 1.73	0.29			
Perfusion MRI						
No	Ref	-	-	Ref	-	-
Yes	0.56	0.39, 0.80	0.001*	0.60	0.41, 0.86	0.006*
Nuclear imaging						
No	Ref	-	-			
Yes	0.73	0.42, 1.28	0.27			

^*^Significant at *p* < 0.05 level

Abbreviations: CI, confidence interval; EQD2, equivalent dose in 2 Gy fractions; Gy, Gray; RU, radiographic uncertainty; ARE, adverse radiation effect; MRI, magnetic resonance imaging

### Diagnosis and management

Data on lesion diagnosis (determined either radiologically or pathologically) and definitive management are shown in Table 3. ARE was diagnosed in 57.0% of lesions, while 42.2% of lesions were diagnosed as progressive tumor. One lesion was ultimately found to be a cavernous malformation upon resection 2.5 years after SRS. A diagnosis was reached radiographically in 73.4% of cases and based on surgical pathology in 26.6%. Of those that were resected, the presumed preoperative diagnosis aligned with surgical pathology in 88.2% of cases (three lesions thought to represent tumor were ultimately diagnosed as ARE and one lesion thought to represent tumor was diagnosed as a cavernous malformation). Management of the nine lesions that had previously been treated to an EQD2 ≥ 100 Gy involved surgery in 44.4% of cases. Surveillance between the time of initial RU and RU resolution involved MR perfusion imaging in 52.2% of cases and PET imaging in 11.7% of cases. Lesions in which MR perfusion was used were larger at the time of initial RU (7.9 cc vs. 4.1 cc, *p* = 0.01) and were more often located in the temporal lobes (24.1% vs. 8.9%, *p* = 0.08) compared to those in which MR perfusion was not used. Of the 34 lesions treated with surgery at the time of intervention, 16 had been imaged with MR perfusion between RU and RU resolution. The interpretation of MR perfusion (i.e., ARE versus tumor progression) was consistent with pathologic findings at surgery in 75% of cases.

**Table 3:** Management at time of RU resolution

Parameter	Frequency of lesions (%)
Diagnosis	
ARE	73 (57.0)
Tumor progression	54 (42.2)
Cavernous malformation	1 (0.78)
Method of diagnosis	
Radiographic	94 (73.4)
Pathologic	34 (26.6)
Parameter	Frequency of patients (%)
Intervention at RU resolution	
No	53 (41.5)
Yes	74 (58.5)
Radiation	22 (17.2)
Surgery	23 (18.0)
Radiation and surgery	11 (8.6)
Systemic therapy	6 (4.7)
Hospice	6 (4.7)
Steroids	5 (3.9)
Clinical trial	1 (0.8)
Neurologic deficit surrounding intervention	28/72 (38.9)

**Fig. 3:**
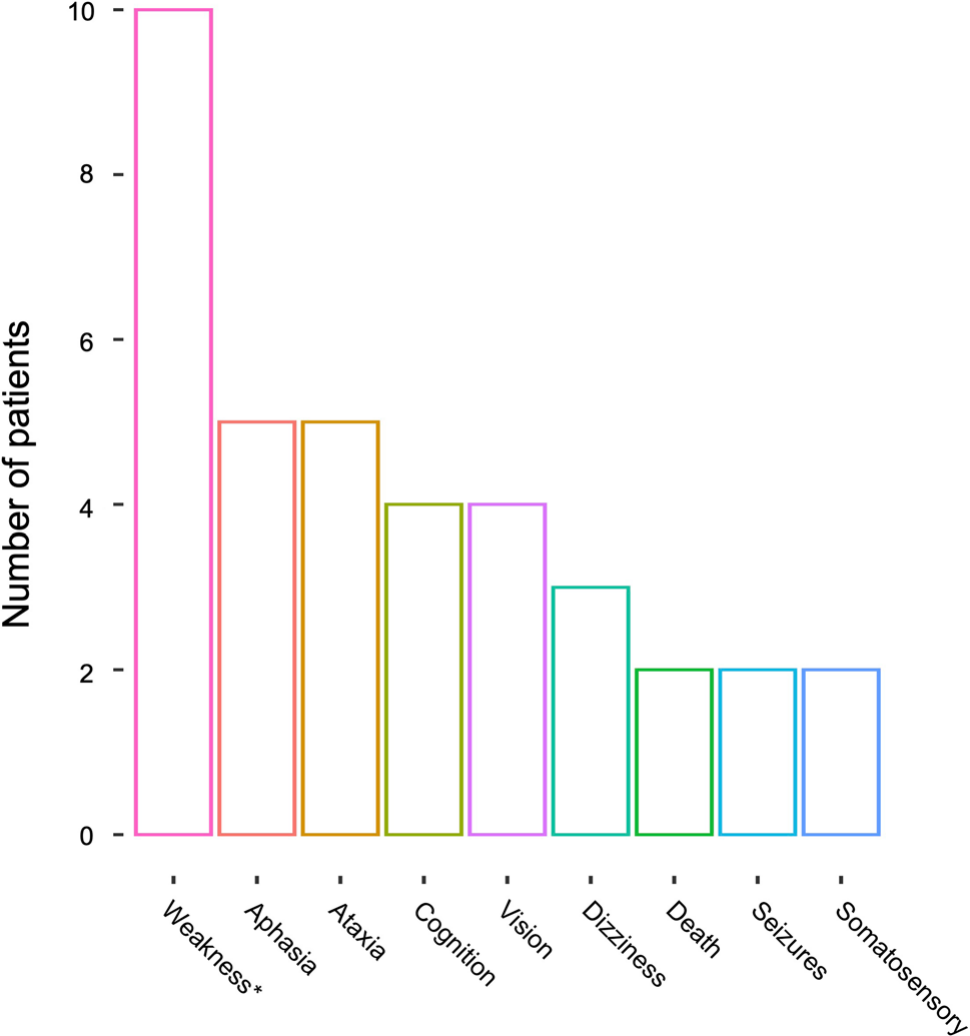
Frequency of neurologic deficits developed surrounding definitive intervention

At the time of RU resolution, 58.5% of patients underwent an intervention, including radiation (17.2%), surgery (18.0%), or a combination of the two (8.6%). Of these, 37.8% developed a neurologic deficit surrounding intervention. Most patients developed these deficits leading up to the intervention due to continued progression of either ARE or tumor; however, two patients developed new deficits immediately after the intervention (one who underwent surgery and another who underwent repeat SRS). The types and frequencies of the neurologic deficits experienced by patients in this cohort are illustrated in Fig. 3. The most common neurologic deficits were weakness, aphasia, and ataxia. Two patients died shortly after RU resolution. One patient, in whom RU resolution took 2.4 months, died from intractable cerebral edema secondary to radiation necrosis. The other patient was ultimately diagnosed with recurrent tumor 6.5 months after initial RU, during which time the lesion grew from 7.35 cc to 30.16 cc. This patient died due to progression of intracranial disease.

## Discussion

While tumor progression and ARE are often radiographically and clinically indistinguishable, their management can be vastly different. Current diagnostic methods are non-specific in this setting despite efforts from the Response Assessment in Neuro-Oncology (RANO) group to standardize treatment response reporting [15]. When RU between progressive disease and ARE arises, patients are frequently monitored clinically and with serial imaging until a diagnosis can be reached. The implications of this RU are crucial to understand as the incidence of brain metastases rises and SRS plays a larger role in their treatment. In this study, we described the management and outcomes among patients with brain metastases presented at our multidisciplinary SRS Tumor Board for RU after SRS. Our findings illustrate the implications of RU and underscore the need for improved diagnostic techniques in this patient population.

While resolution of RU occurred within 3 months of initial RU for most lesions in our study, a large subset was subject to substantial diagnostic delays. Notably, RU resolution took more than 6 months in 25% of our cohort. Differentiation of tumor progression from ARE is notoriously difficult for several reasons. ARE encompasses a heterogeneous group of responses to intracranial radiotherapy ranging from transient, early inflammation, or “pseudoprogression,” to irreversible and sometimes progressive brain necrosis [16]. These entities exhibit marked variation in imaging features, making it challenging to define a characteristic “ARE radiologic signature” that can be applied across patients. Two-thirds of our cohort were receiving systemic therapy at the time of RU, which can influence treated lesions’ appearance on MRI. Treatment effect can also differ depending on class of systemic agent, with several studies demonstrating an increased risk of ARE with capecitabine [10] and tyrosine kinase inhibitors [17]. Moreover, intracranial lesions often exhibit dynamic evolution on imaging after SRS; up to a third will enlarge after treatment [18]. While this typically occurs within 18 months of radiation [10], cases have been reported up to a decade later [19]. This variability is reflected in our cohort, in which the time from SRS to RU development among patients ultimately diagnosed with ARE ranged from 1.1 to 156 months. Finally, the risk factors for development of ARE are incompletely understood and thus have poor specificity for treatment effect. Certainly, they include treatment-related parameters such as radiation dose, volume [20], and concurrent use of systemic therapy [10, 17], but also comprise primary tumor histology [21], age [22], tumor location and microenvironment [16, 23], intrinsic radiosensitivity [24], or most likely, a combination of these factors.

It took less time to resolve RU for lesions that had received multiple prior courses of radiotherapy (i.e., had been treated to a cumulative EQD2 ≥ 100 Gy). Cumulative radiation dose and volume of irradiated brain tissue, specifically V10 Gy and V12 Gy [20], have previously been described as risk factors for ARE. For instance, a review of 435 patients with 2,200 brain metastases treated with Gamma Knife SRS found that prior SRS to the same lesion was the most important predictor of ARE. Specifically, previous radiosurgery was associated with a 20% 1-year risk of symptomatic ARE, compared with only 3% among those with no prior treatment [10]. Our findings imply that radiation treatment history is often considered in diagnostic decision-making between RU and tumor progression. This may influence management in several ways. Progressive or symptomatic lesions may be pushed towards earlier surgery to obtain a pathologic diagnosis given the risks associated with both observation and further radiation. Indeed, 44% of the lesions previously treated to a total EQD2 ≥ 100 Gy in our study were diagnosed surgically, compared with 18% in the larger cohort. Alternatively, asymptomatic lesions that have been treated with multiple prior courses of radiotherapy may be quickly attributed to ARE.

Lesion diameter and volume at the time of RU were also associated with time to resolution of RU. One strategy for distinguishing treatment effect from progressive disease is comparison of the MRI with RU to the original radiation plan. Out-of-field or marginal lesion growth (i.e., growth outside of the area previously treated to 10–12 Gy) is concerning for recurrent tumor, while enlargement within the high-dose treatment area is more convincing for ARE. Small lesions are typically clearly distinguishable as in- or out-of-field, leading to prompt diagnosis and intervention, if appropriate. Alternatively, larger lesions often demonstrate heterogeneous growth [25], requiring observation over multiple imaging cycles before a definitive pattern of evolution can be appreciated. Moreover, larger lesions are more likely to contain a mixture of ARE, progressive disease, and necrotic tumor, further complicating their characterization [26, 27]. We also found that use of MR perfusion imaging portended a shorter time to resolution of RU. MR perfusion is often employed to help distinguish ARE from tumor progression [12, 28]; however, it is not without limitations, particularly for small lesions. This is illustrated in our study, where findings on MR perfusion only correlated with surgical pathology in 75% of cases.

Interestingly, there were a number of clinically relevant factors that were not associated with time to RU resolution. Perhaps most importantly, there was no difference in time to RU resolution between lesions ultimately deemed to represent ARE versus progressive disease, emphasizing the diagnostic challenge central to this study. In the same vein, the presence of symptoms at the time of RU did not correlate with more rapid resolution, likely because ARE can mimic tumor progression clinically [9]. Symptomatic ARE is initially managed with corticosteroids and sometimes bevacizumab, both of which can mitigate the clinical and radiographic signs of radionecrosis [29–31]. In clinical practice, these therapies frequently serve a diagnostic purpose as well, as one would expect recurrent tumor to remain stable and ARE to rapidly regress after their administration. Despite this, we did not find that lesions treated with steroids or bevacizumab at the time of RU were resolved more quickly. This may have been because these therapies are also used to relieve symptoms of recurrent brain metastases, and both are also associated with reversal of radiographic findings of tumor progression [8, 32]. Interpretation of imaging after administration of steroids or bevacizumab is therefore complex, and our findings suggest that they may not always facilitate differentiation of progressive disease from ARE.

Strikingly, almost 40% of patients who underwent a definitive intervention upon RU resolution developed a new neurologic deficit surrounding intervention. These included symptoms such as weakness, aphasia, visual deficits, and cognitive changes, all of which can have a profound impact on functional status and quality of life. While we are unable to determine whether these could have been prevented with earlier diagnosis, most deficits developed due to continued progression of ARE or tumor, and it is reasonable to assume that they could have been mitigated or avoided altogether with earlier intervention. Indeed, we found that median lesion size increased by 25% and median lesion volume almost doubled between RU and consensus diagnosis. This was accompanied by a significantly higher proportion of patients experiencing edema causing mass effect at the time of resolution. Larger lesion size is also a known risk factor for treatment-related complications after surgery [33, 34] and radiotherapy [9], which occurred in two patients who underwent definitive intervention at the time of RU resolution. Two patients in our cohort died shortly after resolution of RU. One developed intractable intracranial edema due to radiation necrosis, ultimately leading to their death 2.4 months after initial RU. Another patient died from progressive disease 6.5 months after RU, during which time their tumor quadrupled in size. While these represent extreme examples, our findings suggest that a large proportion of patients develop serious, potentially irreversible deficits due to diagnostic delay. Even with timely intervention, diagnostic uncertainty raises the potential for inappropriate treatment, such as re-reirradiation or withdrawal of effective systemic therapy in a patient who has ARE but is thought to have recurrent tumor. It is important to note that resolution of RU is often based on imaging alone without pathologic confirmation [16], as was the case in 73% of lesions in this study. Thus, there is a real possibility of misdiagnosis. This is highlighted in our cohort, where the presumed preoperative diagnosis among lesions that were surgically resected only aligned with pathologic findings in 88% of cases. This can lead to treatment error, unnecessary toxicity, and compromised outcomes.

RU is a well-recognized problem in the field of Neuro-Oncology [8, 15, 16], and efforts to develop more effective diagnostic tools to distinguish ARE from tumor progression are currently underway [35]. LITT, a minimally invasive surgical technique that uses heat to ablate tissue, can play both diagnostic and therapeutic roles in the management of RU. Indications for LITT have rapidly expanded in recent years and now include both treatment of in-field metastatic recurrences and ARE [36]. LITT has also demonstrated superior efficacy to bevacizumab for symptomatic radiation necrosis in retrospective series [37, 38]. Nevertheless, LITT may not always be feasible, particularly in lesions located in eloquent brain regions. Non-invasive diagnostic techniques have also been explored to address RU. These have included using standard MRI sequences such as T1/T2 matching [11], apparent diffusion coefficient ratios [39], and time-dependent changes in lesion morphology after contrast administration [40], although these are infrequently used in the clinical setting. As discussed above, MR perfusion is often employed when conventional MRI sequences are inconclusive, and multiple groups have explored using standard cutoffs for metrics such as relative cerebral blood volume or peak height to distinguish recurrent tumor from treatment effect [12, 28]. MR spectroscopy [41–43], which analyzes tissue metabolites is another MR technique that has been employed to improve diagnostic certainty in this situation. Historically, single-photon emission computed tomography (SPECT)^44^, which detects the distribution of radioactive tracers such as thallium-201 or technetium-99, was the nuclear medicine technique of choice for the study of indeterminate brain lesions. More recently, amino acid PET imaging has been employed in this scenario and has demonstrated high diagnostic accuracy in identifying tumor progression [45, 46]. Indeed, RANO cites level 2 evidence for amino acid PET in this setting, although most studies have comprised retrospective, single-center analyses without histopathologic confirmation [47]. While a variety of amino acid radiotracers have been examined, including ^11^C-MET, ^18^F-DOPA, ^18^F-FET, and ^18^F-fluciclovine, their regular use is currently limited by availability, cost, and regulatory approval [48]. Notably, only 11.7% of lesions in our study were assessed with nuclear imaging, highlighting its limited use even within a large, academic institution. Most recently, machine learning-based analyses of radiomics signatures have been increasingly explored in patients with RU after intracranial radiation [49]. This approach extracts radiographic features from a variety of imaging sequences and modalities to build predictive models that can aid in diagnosis and management. These models frequently outperform radiologists in identifying radiation necrosis [50, 51]; however, further validation is needed in large, heterogeneous populations across multiple sites before they can be incorporated into clinical practice. Ultimately, RANO guidelines state that there is insufficient evidence to support any one of these modalities [15]. Instead, use of clinical judgment with a combination of these techniques is recommended, potentially across several imaging cycles if a diagnosis cannot be reached at any one timepoint.

This study has several limitations, one of which is its lack of histopathologic confirmation at the time of resolution for the majority of lesions. Our findings describe factors associated with time to RU resolution, which may or may not have been based on a correct diagnosis in patients diagnosed radiographically. Nevertheless, our results reflect the reality of RU resolution in the clinical setting and describe the consequences of current management practices. Other limitations are related to this study’s retrospective nature. Patient-specific factors such as lack of social support or transportation barriers that may have contributed to prolonged diagnosis were not captured in this study. Similarly, assessment of patient symptoms upon RU and intervention was based on the medical record, which may have introduced bias in our results. However, complete radiation records and imaging at each timepoint were available for the vast majority of lesions, which allowed the study authors to independently measure lesion volumes over time rather than relying on radiology reports.

To our knowledge, we present the first analysis of the consequences of RU between ARE and tumor progression among patients who have undergone SRS for brain metastases. In a multidisciplinary setting at a large, academic medical center, a quarter of patients experienced delays in management of at least 6 months, during which time lesions enlarged and more often led to intracranial edema. Clinical factors such as radiation history, lesion size, and use of MR perfusion influenced time to RU resolution; however, these may be subject to error when used for diagnostic purposes. Finally, a striking number of patients developed neurologic deficits surrounding intervention, including two patients who died due to lesion progression. Oncology care teams will increasingly be faced with this diagnostic dilemma as survival among patients with brain metastases continues to improve. Our findings highlight the critical need for more effective, clinically feasible diagnostic tools in this population.

## Data Availability

The data analyzed in this study are available upon request to the corresponding author.

## References

[1] Gondi V, Bauman G, Bradfield L (2022). Radiation Therapy for Brain Metastases: An ASTRO Clinical Practice Guideline. Pract Radiat Oncol.

[2] Suh JH (2010). Stereotactic radiosurgery for the treatment of brain metastases. N Engl J Med.

[3] Brown PD, Jaeckle K, Ballman KV (2016). Effect of radiosurgery alone vs radiosurgery with whole brain radiation therapy on cognitive function in patients with 1 to 3 brain metastases a randomized clinical trial. JAMA - J Am Med Assoc.

[4] Chang EL, Wefel JS, Hess KR (2009). Neurocognition in patients with brain metastases treated with radiosurgery or radiosurgery plus whole-brain irradiation: a randomised controlled trial. Lancet Oncol.

[5] Aoyama H, Shirato H, Tago M (2006). Stereotactic radiosurgery plus whole-brain radiation therapy vs stereotactic radiosurgery alone for treatment of brain metastases: A randomized controlled trial. JAMA - J Am Med Assoc.

[6] Yamamoto M, Serizawa T, Shuto T (2014). Stereotactic radiosurgery for patients with multiple brain metastases (JLGK0901): a multi-institutional prospective observational study. Lancet Oncol.

[7] Eisele S, Dietrich J (2015). Cerebral radiation necrosis: diagnostic challenge and clinical management. Rev Neurol.

[8] Aizer AA, Lamba N, Ahluwalia MS (2022). Brain metastases: A Society for Neuro-Oncology (SNO) consensus review on current management and future directions. Neuro Oncol.

[9] Minniti, G., Clarke, E., Lanzetta, G. et al (2021) Stereotactic radiosurgery for brain metastases: analysisof outcome and risk of brain radionecrosis. Radiat Oncol 6:48 . 10.1186/1748-717X-6-48 10.1186/1748-717X-6-48 PMC3108308 21575163

[10] Sneed PK, Mendez J, Vemer-Van Den Hoek JGM (2015). Adverse radiation effect after stereotactic radiosurgery for brain metastases: Incidence, time course, and risk factors. J Neurosurg..

[11] Kano H, Kondziolka D, Lobato-Polo J, Zorro O, Flickinger JC, Lunsford LD (2010). T1/T2 matching to differentiate tumor growth from radiation effects after stereotactic radiosurgery. Neurosurgery.

[12] Mitsuya K, Nakasu Y, Horiguchi S (2010). Perfusion weighted magnetic resonance imaging to distinguish the recurrence of metastatic brain tumors from radiation necrosis after stereotactic radiosurgery. J Neurooncol.

[13] Kamada K, Houkin K, Abe H, Sawamura Y, Kashiwaba T (1997). Differentiation of cerebral radiation necrosis from tumor recurrence by proton magnetic resonance spectroscopy. Neurol Med Chir.

[14] Li H, Deng L, Bai HX (2018). Diagnostic Accuracy of Amino Acid and FDG-PET in Differentiating Brain Metastasis Recurrence from Radionecrosis after Radiotherapy: A Systematic Review and Meta-Analysis. Am J Neuroradiol.

[15] Lin NU, Lee EQ, Aoyama H (2015). Response assessment criteria for brain metastases: Proposal from the RANO group. Lancet Oncol.

[16] Winter SF, Loebel F, Loeffler J (2019). Treatment-induced brain tissue necrosis: A clinical challenge in neuro-oncology. Neuro Oncol.

[17] Kim JM, Miller JA, Kotecha R (2017). The risk of radiation necrosis following stereotactic radiosurgery with concurrent systemic therapies. J Neurooncol.

[18] Patel TR, McHugh BJ, Bi WL, Minja FJ, Knisely JPS, Chiang VL (2011). A comprehensive review of MR imaging changes following radiosurgery to 500 brain metastases. Am J Neuroradiol.

[19] Tofilon PJ, Fike JR (2000). The radioresponse of the central nervous system: A dynamic process. Radiat Res.

[20] Blonigen BJ, Steinmetz RD, Levin L, Lamba MA, Warnick RE, Breneman JC (2010). Irradiated Volume as a Predictor of Brain Radionecrosis After Linear Accelerator Stereotactic Radiosurgery. Int J Radiat Oncol Biol Phys.

[21] Miller JA, Bennett EE, Xiao R (2016). Association Between Radiation Necrosis and Tumor Biology After Stereotactic Radiosurgery for Brain Metastasis. Int J Radiat Oncol Biol Phys.

[22] Kerschbaumer J, Demetz M, Krigers A, Nevinny-Stickel M, Thomé C, Freyschlag CF (2021). Risk factors for radiation necrosis in patients undergoing cranial stereotactic radiosurgery. Cancers (Basel).

[23] Jarosz-Biej M, Smolarczyk R, Cichoń T, Kułach N (2019). Tumor microenvironment as a “game changer” in cancer radiotherapy. Int J Mol Sci.

[24] Raaphorst GP, Malone S, Alsbeih G, Souhani L, Szumacher E, Girard A (2002). Skin fibroblasts in vitro radiosensitivity can predict for late complications following AVM radiosurgery. Radiother Oncol.

[25] Wen J, Tan AP, Yong HRC, Wong YLJ (2018). Delayed radiation necrosis and evolution of its imaging features over time: An illustrative case report. J Oncol Pract.

[26] Dequesada IM, Quisling RG, Yachnis A, Friedman WA (2008). Can standard magnetic resonance imaging reliably distinguish recurrent tumor from radiation necrosis after radiosurgery for brain metastases? A radiographic-pathological study. Neurosurgery.

[27] Mullins ME, Barest GD, Schaefer PW, Hochberg FH, Gonzalez RG, Lev MH (2005). Radiation necrosis versus glioma recurrence: Conventional MR imaging clues to diagnosis. Am J Neuroradiol.

[28] Barajas RF, Chang JS, Sneed PK, Segal MR, McDermott MW, Cha S (2009). Distinguishing recurrent intra-axial metastatic tumor from radiation necrosis following gamma knife radiosurgery using dynamic susceptibility- weighted contrast-enhanced perfusion MR imaging. Am J Neuroradiol.

[29] Tye K, Engelhard HH, Slavin KV (2014). An analysis of radiation necrosis of the central nervous system treated with bevacizumab. J Neurooncol.

[30] Rahmathulla G, Marko NF, Weil RJ (2013). Cerebral radiation necrosis: A review of the pathobiology, diagnosis and management considerations. J Clin Neurosci.

[31] Yoritsune E, Furuse M, Kuwabara H (2014). Inflammation as well as angiogenesis may participate in the pathophysiology of brain radiation necrosis. J Radiat Res.

[32] Berghoff AS, Breckwoldt MO, Riedemann L (2020). Bevacizumab-based treatment as salvage therapy in patients with recurrent symptomatic brain metastases. Neuro-Oncology Adv.

[33] Ferroli P, Broggi M, Schiavolin S (2015). Predicting functional impairment in brain tumor surgery: The Big Five and the Milan Complexity Scale. Neurosurg Focus.

[34] McPherson CM, Warnick RE (2004). Results of contemporary surgical management of radiation necrosis using frameless stereotaxis and intraoperative magnetic resonance imaging. J Neurooncol.

[35] Mayo ZS, Halima A, Broughman JR (2023). Radiation necrosis or tumor progression? A review of the radiographic modalities used in the diagnosis of cerebral radiation necrosis. J Neurooncol.

[36] Holste KG, Orringer DA (2020). Laser interstitial thermal therapy. Neuro-Oncology Adv.

[37] Sujijantarat N, Hong CS, Owusu KA (2020). Laser interstitial thermal therapy (LITT) vs. bevacizumab for radiation necrosis in previously irradiated brain metastases. J Neurooncol..

[38] Chan M, Tatter S, Chiang V et al (2023) Neuro-Oncology Advances proven radiation necrosis in radiographically recurrent 5(March):1-12 10.1093/noajnl/vdad031 PMC10129388 37114245

[39] Hein PA, Eskey CJ, Dunn JF, Hug EB (2004). Diffusion-Weighted Imaging in the Follow-up of Treated High-Grade Gliomas: Tumor Recurrence versus Radiation Injury. Am J Neuroradiol.

[40] Wagner S, Lanfermann H, Eichner G, Gufler H (2017). Radiation injury versus malignancy after stereotactic radiosurgery for brain metastases: Impact of time-dependent changes in lesion morphology on MRI. Neuro Oncol.

[41] Sundgren PC (2009). MR spectroscopy in radiation injury. Am J Neuroradiol.

[42] Rock JP, Scarpace L, Hearshen D (2004). Associations among Magnetic Resonance Spectroscopy, Apparent Diffusion Coefficients, and Image-guided Histopathology with Special Attention to Radiation Necrosis. Neurosurgery.

[43] Wang ZJ, Ohliger MA, Larson PEZ (2019). Hyperpolarized 13C MRI: State of the art and future directions. Radiology.

[44] Furuse M, Nonoguchi N, Yamada K (2019). Radiological diagnosis of brain radiation necrosis after cranial irradiation for brain tumor: A systematic review. Radiat Oncol.

[45] Cicone F, Minniti G, Romano A (2015). Accuracy of F-DOPA PET and perfusion-MRI for differentiating radionecrotic from progressive brain metastases after radiosurgery. Eur J Nucl Med Mol Imaging.

[46] Tomura N, Kokubun M, Saginoya T, Mizuno Y, Kikuchi Y (2017). Differentiation between Treatment-Induced Necrosis and Recurrent Tumors in Patients with Metastatic Brain Tumors: Comparison among 11C-Methionine-PET, FDG-PET, MR Permeability Imaging, and MRI-ADC - Preliminary Results. Am J Neuroradiol.

[47] Galldiks N, Langen KJ, Albert NL (2019). PET imaging in patients with brain metastasis—report of the RANO/PET group. Neuro Oncol.

[48] Heinzel A, Müller D, Yekta-Michael SS (2017). O-(2–18F-fluoroethyl)-L-tyrosine PET for evaluation of brain metastasis recurrence after radiotherapy: An effectiveness and cost-effectiveness analysis. Neuro Oncol.

[49] Nowakowski A, Lahijanian Z, Panet-Raymond V (2022). Radiomics as an emerging tool in the management of brain metastases. Neuro-Oncology Adv.

[50] Hettal L, Stefani A, Salleron J (2020). Radiomics method for the differential diagnosis of radionecrosis versus progression after fractionated stereotactic body radiotherapy for brain oligometastasis. Radiat Res.

[51] Peng L, Parekh V, Huang P (2018). Distinguishing true progression from radionecrosis after stereotactic radiation therapy for brain metastases with machine learning and radiomics. Int J Radiat Oncol Biol Phys.

